# NARROWING THE DISPARITIES GAP IN LTSS: POLICY AND PRACTICE

**DOI:** 10.1093/geroni/igac059.592

**Published:** 2022-12-20

**Authors:** Rita Choula

## Abstract

Access to and the quality of long-term supports and services (LTSS) are not equitable for all older Americans. Disparities have been documented qualitatively and quantitatively for marginalized racial and ethnic communities and LGBTQI+ communities but the specific causes of gaps in equity differ by community, locality, and state. To be effective, policy solutions must be grounded in the lived experiences of Black, Latino, Asian American and Pacific Islander, and LGBTQI+ older adults in those communities. This symposium showcases how community-based research can be employed to understand the root causes of inequities in LTSS access and care affecting older adults of color and LTGBTQI+-identifying older adults and presents community-grounded policy solutions to remedy those inequities. Papers 1 and 2 use participatory research methods to understand barriers to equitable LTSS care access and quality and develop locally grounded solutions to those barriers. Caldera (Paper 1) shares results from a study of Cook County, IL nursing home residents and their caregivers focused on racial and ethnic disparities in access to and experiences with nursing home care. Hado (Paper 2) presents findings from research in Georgia and New York examining disparities in access to and experiences with HCBS for older racial and ethnic and LGBTQI+ communities. Fashaw-Walters (Paper 3) discusses the how systemic racism is at the root of inequities in LTSS access for communities of color and shares actionable recommendations aimed at ending racial and ethnic inequities in LTSS policies.

